# 支气管肺类癌临床和病理学特点的回顾性研究

**DOI:** 10.3779/j.issn.1009-3419.2010.06.005

**Published:** 2010-06-20

**Authors:** 洪亮 廖, 慧兰 饶, 旭 张, 勇斌 林, 明然 解, 剑华 傅, 浩 龙, 铁华 戎, 鹏 林

**Affiliations:** 1 510060 广州，华南肿瘤学国家重点实验室，中山大学肿瘤防治中心胸科 State Key Laboratory of Oncology in South China, Department of Thoracic Surgery, Cancer Center of Sun Yat-sen University, Guangzhou 510060, China; 2 510060 广州，中山大学肿瘤防治中心病理科 Department of Pathology, Cancer Center of Sun Yat-sen University, Guangzhou 510060, China

**Keywords:** 支气管肺类癌, 典型类癌, 非典型类癌, Bcl-2蛋白, Ki-67抗原, Bronchopulmonary carcinoid, Typical carcinoid, Atypical carcinoid, B cell lymphoma-2 protein, Ki-67 Antigen

## Abstract

**背景与目的:**

支气管肺类癌（bronchopulmonary carcinoid, BPC）占全部肺原发性恶性肿瘤不到2%，相关研究报道较少，本研究拟分析BPC的总体及其两个亚型——典型类癌（typical carcinoid, TC）和不典型类癌（atypical carcinoid, AC）的临床、病理学特点。

**方法:**

回顾性分析中山大学肿瘤防治中心1994年1月-2009年6月收治的28例BPC的临床资料，调取可再行切片的病理蜡块，重新切片染色及病理玻片会诊，分析BPC的总体及其亚型的临床特征和相关免疫组化指标与预后的关系。

**结果:**

全部28例患者的总体5年生存率为56%，TC的5年生存率为70%，AC为41%，单因素分析示TNM分期（*P*=0.037)、有无淋巴结转移（*P*=0.001）、Ki-67核阳性数是否大于5%（*P*=0.009）是BPC总体的预后因素。相关性分析示BPC亚型与CD99、Bcl-2及Ki-67的表达具有相关性（*P*值分别0.017、0.043、0.033）。20例行肺癌根治性手术患者的5年生存率为73%，TC的5年生存率为83%，AC为57%。单因素分析示BPC亚型（*P*=0.013）、术后有无淋巴结转移（*P*=0.004)、Ki-67核阳性数是否大于5%（*P*=0.006）、TNM分期（*P*=0.047）是行肺癌根治性手术患者的预后因素。肿瘤复发（*n*=4）与Ki-67核阳性的表达和Bcl-2表达具有相关性（*P*值分别为0.027、0.045）。

**结论:**

BPC是预后较好的肺原发性恶性肿瘤，Ki-67、Bcl-2的高表达是提示BPC复发及预后不良的影响因素，TNM分期是影响预后的独立因素，行根治性手术是主要的治疗手段。

## Abstract (en)

**Background and objective:**

Bronchopulmonary carcinoid (BPC) account for less than 2% of all primary lung malignant tumors, but few related studies were reported. The aim of this study is to analyze this rare disease's clinicopathological characteristics.

**Methods:**

The clinical data of 28 patients with BPC in Cancer Center of Sun Yat-sen University, from January 1994 to June 2009, were enrolled into retrospective analysis. First, the corresponding paraffin blocks reexamined, slice up and stained, multiple pathologists re-consulted, and its subsets (typical carcinoid, TC; atypical carcinoid, AC) defined. Second, the clinical characteristics and immunohistochemical markers and its relationship with prognosis were analyzed.

**Results:**

First, the 5-year survival for overall and TC, AC was 56% and 70%, 41% respectively in 28 cases. The markers CD99, Bcl-2 and Ki-67 expression correlated significantly with the BPC subsets (*P*=0.017, *P*=0.043, and *P*=0.033 respectively). Further univariate analysis revealed that advanced TNM staging (*P*=0.037), lymph node metastasis (*P*=0.001) and Ki-67 nucleolus's positive expression (*P*=0.009) are poor prognostic factors. Second, the overall, TC, AC 5-year survival rate was 73%, 83%, 57% respectively in 20 cases underwent the radically surgical resection. Further univariate analysis revealed that AC subset (*P*=0.013), lymph node metastasis (*P*=0.004) and Ki-67 nucleolus's positive expression (*P*=0.006), advanced TNM staging (*P*=0.047) are poor prognostic factors in this 20 cases. Third, as univariate analysis, local recurrence and metastasis (*n*=4) correlate significantly with Ki-67 nucleolus's and Bcl-2 positive expression (*P*=0.027, 0.045, respectively).

**Conclusion:**

The prognosis of BPC was better than other types of primary lung cancer. Ki-67, bcl-2 high expression and advanced TNM staging are the poor recurrence and prognostic factors of BPC. The radical surgery remains the treatment of choice for resectable candidates in BPC as NSCLC.

支气管肺类癌(bronchopulmonary carcinoid, BPC)占全部肺原发性恶性肿瘤不到2%^[1, 2]^。根据其临床特征和预后，Travis等^[3, 4]^提出了目前被WHO(2004)和IASLC(2009)采用的分类标准：根据细胞分化、核分裂强度及是否有坏死，WHO(2004)把支气管肺类癌分为典型类癌(typical carcinoid, TC)和不典型类癌(atypical carcinoid, AC)，其中无坏死以及每个高倍镜下有丝分裂像少于2个的定义为TC，而AC是每个高倍视野下有2-10个有丝分裂，说明有坏死。

神经内分泌标志物(NSE、CgA、Syn、CD56等)^[5]^一般在BPC中表达呈阳性，特别是在TC中表达呈强阳性。TTF1表达于甲状腺及肺上皮中，对于在TC和AC中是否表达尚存争议^[6-8]^。S-100也是一个常用的神经内分泌标志物，通常表达于肿瘤的支持细胞中^[9]^。Ki-67和Bcl-2分别是评估细胞增殖及程序性死亡失调的指标；Ki-67强阳性及Bcl-2高表达是预后不良的指标^[10-12]^。

本文回顾性分析中山大学肿瘤防治中心1994年1月-2009年6月收治的28例BPC患者临床资料，分析其总体及亚型的临床和病理免疫标志物特征及影响预后的因素。

## 1 资料和方法

### 1.1 研究方法

本文复习中山大学肿瘤防治中心近15年收治的28例BPC病例资料，调取全部28例病理玻片重新会诊，重新调取22例可行再切片的蜡块，行HE染色及肿瘤免疫组化检测(CK、NSE、CgA，Syn、CD56、TTF1、S-100、CD99、Ki-67、Bcl-2)，明确BPC病理亚型及免疫组化结果。

### 1.2 随访及预后

生存期即自确诊之日至末次随访日或死亡时间，随访截止至2009年12月，生存期最短5个月、最长188个月，平均52.8个月。死亡9例(TC 3例，AC 6例)。行肺癌根治术20例，术后复发及转移4例(TC 1例，AC 3例)。

### 1.3 统计学分析

用统计软件SPSS 16.0作为统计分析工具，采用*Frequencies*、卡方检验、*Fisher*确切概率法、*Mann-Whitney U*检验、*Kaplan-Meier*法计算生存率，*Log-rank*法进行生存率显著性检验，*Cox*比例风险回归模型进行单因素及多因素分析。以*P* < 0.05为差异有统计学意义。

## 2 结果

### 2.1 一般临床特征

全组患者12例为体检发现(12/28)，占42.9%，常见首发症状是咳嗽，占39.3%(11/28)，其他的症状有咯血丝痰(*n*=02)、胸痛(*n*=02)、发热(*n*=01)、头晕(*n*=01)等。无一例并发类癌综合征。总体患者肿瘤大小≥30 mm占53.6%。TC与AC大小均数分别为40.78 mm与36.33 mm，两组间肿瘤大小无统计学差异(*P*=0.600)(表 1)。

**1:** 28例支气管肺类癌的一般临床特征及TNM分期（UICC分期2009） General clinical characteristics and TNM staging (UICC Staging 2009) of 28 bronchopulmonary carcinoid

	Total	TC	AC
Sex (male/female)	17/11	10/6	7/12
Mean age (years)	43.0±12.5	42.9±13.3	43.1±11.8
Tumor size (mm)	38.88±21.69	40.78±25.82	36.33±15.06
Tumor location			
Central/Peripheral	18/10	11/5	7/5
TNM staging	28	16	12
Ⅰa	7 (25.00%)	6 (37.5%)	1 (8.33%)
Ⅰb	8 (28.57%)	4 (25%)	4 (33.33%)
Ⅱa	3 (10.71%)	1 (6.25%)	2 (16.67%)
Ⅱb	3 (10.71%)	3 (18.75%)	0
Ⅲb	1 (3.57%)	0	1 (8.33%)
Ⅳ	6(21.43%)	2(12.50%)	4 (33.33%)
TC: typical carcinoid; AC: atypical carcinoid.			

### 2.2 病理诊断检查

24例行支气管纤维镜检查：8例镜下未见异常(8/24)，16例镜下见肿物并行支纤镜检查活检，4例病理诊断为神经内分泌癌，占25%(4/16)。另外4例未行支气管纤维镜检查：其中2例行经皮肺穿刺活检，2例行淋巴结活检确诊(1例纵隔镜淋巴结活检，1例颌下淋巴结活检)。

### 2.3 治疗方法

22例行手术治疗：其中20例行肺癌根治术，2例行肺楔形切除术；6例行姑息化疗或加放疗。

### 2.4 BPC亚型及TNM分期

根据肺肿瘤的WHO的分类标准(2004)，16例为TC(16/28)，占57.14%，12例为AC(12/28)，占42.86%(图 1)。根据UICC TNM分期(2009)分期(表 1)，本组病例Ⅰ、Ⅱ、Ⅳ期各占53%、21%、21%，仅1例为Ⅲ期。

**1:**
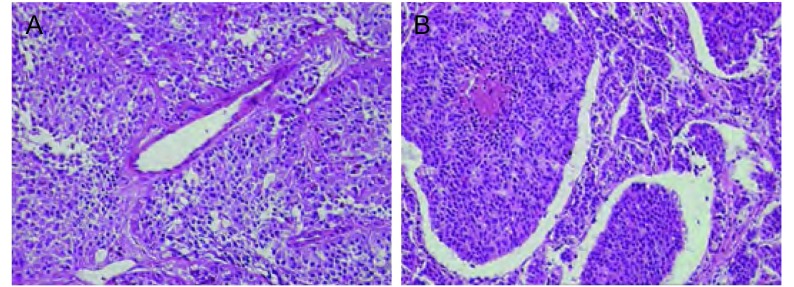
支气管类癌细胞器结构的表现（HE，×200）。A：TC；B：AC。 The organoid pattern of bronchopulmonary carcinoid (HE, ×200). A: TC; B: AC.

### 2.5 免疫组化结果

重新调取22例可行再切片的蜡块，行相关肿瘤免疫组化检测(CK、NSE、CgA，Syn、CD56、TTF1、S-100、CD99、Ki-67、Bcl-2)(图 2)。结合既往免疫组化结果分析其病理临床特点，其中Ki-67用细胞的百分阳性率表示，核阳性表达的中位数为2%，其它指标用阳性及阴性表示(表 2)。

**2:**
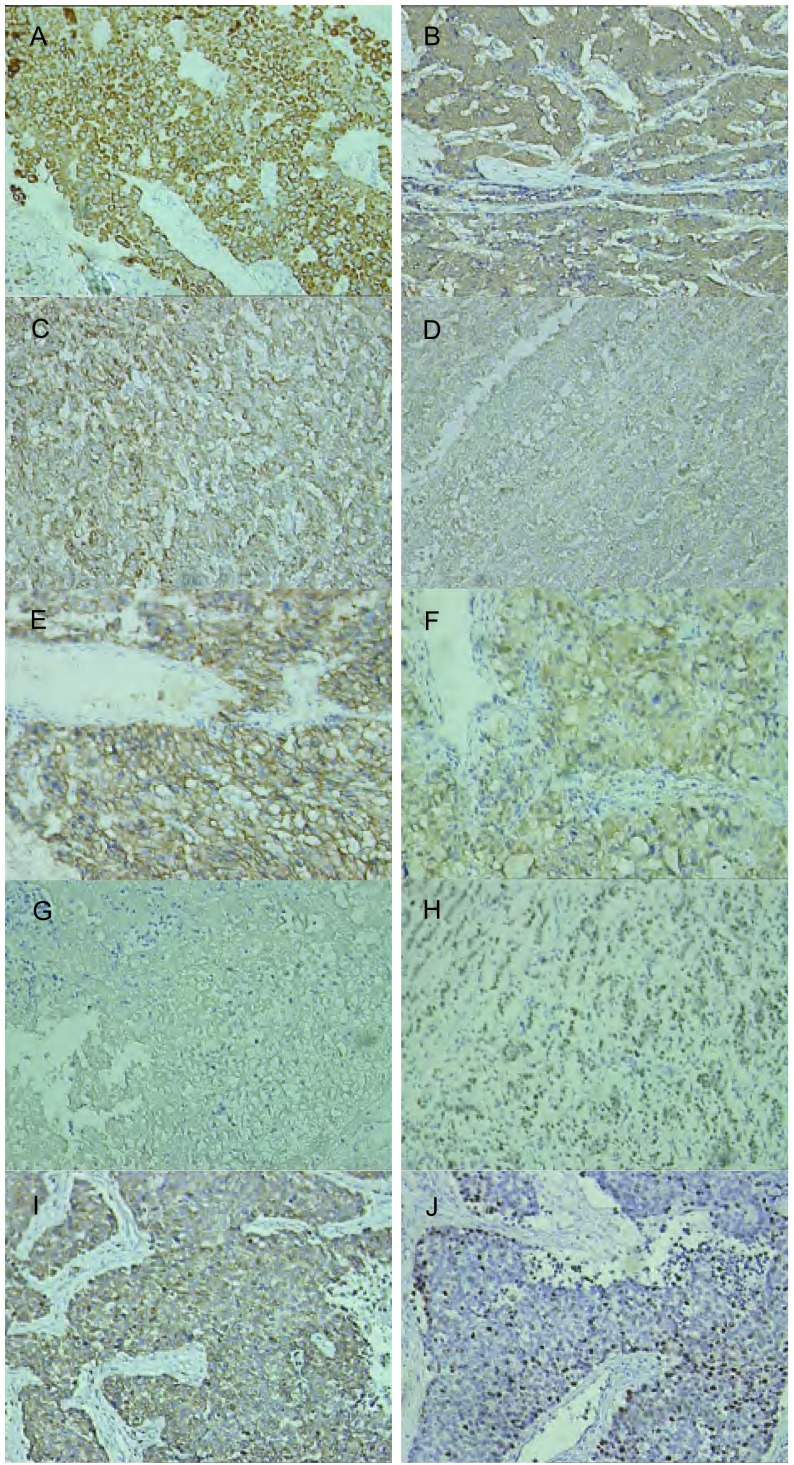
支气管肺类癌在不同免疫组化指标中细胞器结构的阳性表现（×200）。A：CK；B：NSE；C：CgA；D：Syn；E：CD56；F：S-100蛋白；G：CD99；H：TTF-1；I：Bcl-2；J：Ki-67。 Positive expression of different organoid pattern of bronchopulmonary carcinoid are shown by staining for various immunohistochemical marker (×200). A: CK; B: NSE; C: CgA; D: Syn; E: CD56; F: S-100; G: CD99; H: TTF-1; I: Bcl-2; J: Ki-67.

**2:** 28例支气管肺类癌的免疫组化指标的总体阳性率及亚型（TC/AC）的阳性率和TC与AC间统计学比较 The total and subtype (TC/AC) positive ratio and statistical comparisons of IMC marker for TC and AC of 28 bronchopulmonary carcinoid

	Total positive ratio (%)	TC/AC positive ratio (%)	*P*
CK	88.5	86.7/90.7	1.000
NSE	92.3	86.7/100	0.492
CgA	74.1	75.0/72.7	1.000
Syn	96.2	93.8/100	1.000
CD56	87.5	92.9/80.0	0.550
S100	44.0	35.7/54.5	1.000
TTF1	40.0	40.0/40.0	0.435
CD99	40.9	16.7/70	0.027
Bcl-2	22.3	8.3/50	0.043

CD99、Bc l -2及Ki -67在两亚型中的表达有统计学差异，其中CD99(OR=11.667, 95%CI: 1.527-89.121, *Fisher's Exact Test*, *P*=0.017)，Bcl-2(OR=27.5, 95%CI: 1.996-378.837, *Fisher's Exact Test*, *P*=0.043)，Ki-67总体核阳性表达的中位数为2%(TC和AC在表达上有明显的差异，*Mann-Whitney U*，*P*=0.033)。其它标志物未见统计学差异。

### 2.6 预后分析

全组病例5年生存率为56%，其中TC为70%，AC为41%。单因素分析示BPC亚型(*Log-rank*, *Mantel-Cox*, *P*=0.093)、TNM分期(*Log-rank*, *Mantel-Cox*, *P*=0.037)，有无淋巴结转移(*Log-rank*, *Mantel-Cox*, *P*=0.001)、Ki-67核阳性数是否大于5%(*P*=0.009)与预后相关(图 3)。而其它因素：年龄(是否大于60岁)、性别、肿瘤大小(是否大于3 cm)、肿瘤位置(中央型及周围型)、Bcl-2的表达与预后无关。

**3:**
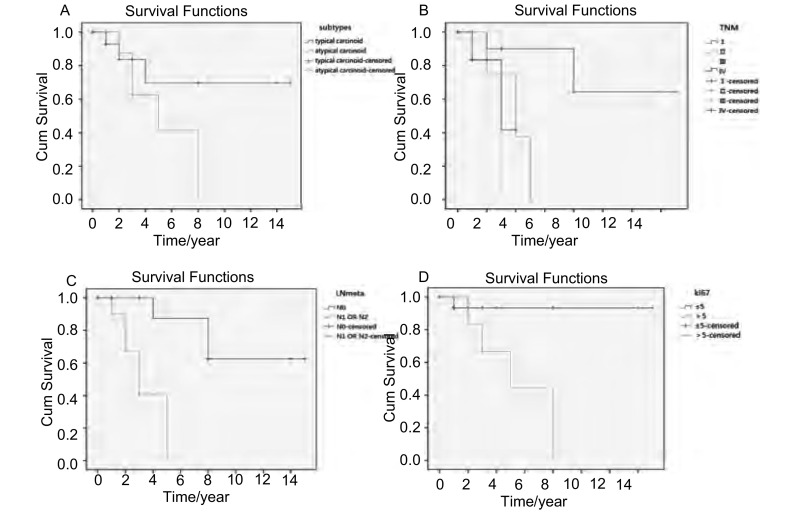
全部患者不同变量的生存曲线。A：BPC亚型；B：TNM分期；C：淋巴结转移；D：KI-67核阳性数是否小于5%。 The survival curves of different variables of the total cases. A: BPC subtypes; B: TNM stage; C: lymph node metastasis; D: The cutoff of 5% of ki67-positive nuclei.

20例行肺癌根治性手术者5年生存率为73%，其中TC为83%，AC为57%。单因素分析示，BPC亚型(*Log-rank*, *Mantel-Cox*, *P*=0.013)、TNM分期(*Log-rank*, *Mantel-Cox*, *P*=0.047)，有无淋巴结转移(*Log-rank*, *Mantel-Cox*, *P*=0.004)、Ki-67核阳性数是否大于5%(*Log-rank*, *Mantel-Cox*, *P*=0.006)与预后相关(图 4)。其他因素：年龄(是否大于60岁)、性别、肿瘤大小(是否大于3 cm)、肿瘤位置(中央型及周围型)、Bcl-2的表达与预后无关；4例分别于术后74、72、18、35个月分别出现肺内、肺内及肝、胸壁肋骨及胸椎转移、肝转移；相关性分析示肿瘤复发与ki-67核阳性(*Mann-Whitney U*, *P*=0.027)、bcl-2(*Fisher's Exact Test*, *P*=0.045)的表达，有统计学差异；与BPC亚型(TC和AC，*Fisher's Exact Test*, *P*=0.101)、年龄(是否大于60岁)、性别、肿瘤大小(是否大于3 cm)、肿瘤位置(中央型及周围型)、是否淋巴结转移无相关。

**4:**
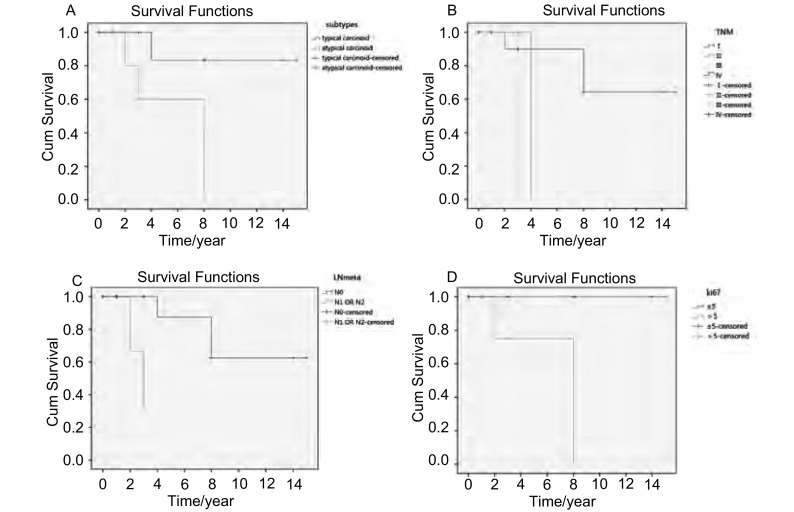
行根治性患者不同变量的生存曲线。A：BPC亚型；B：TNM分期；C：淋巴结转移；D：KI-67核阳性数是否大于5%。 The survival curves of different variables of the adically surgical resection cases. A: BPC subtypes; B: TNM stage; C: lymph node metastasis; D: The cutoff of 5% of ki67-positive nuclei.

## 3 讨论

### 3.1 临床特征

有关BPC的报道中男女发病的比率及其临床特征差异较大，本研究中男女比率17:11，与Fink^[13]^研究中男女比率基本一致。平均年龄为43.0岁，TC与AC的平均年龄分别42.9岁与43.1岁，两者之间无明显差异，与Rugge^[12]^报道基本一致，而Fink^[1]^研究中AC比TC的平均年龄大6岁。支气管肺类癌的原发灶一般比非小细胞肺癌(non-small cell lung cancer, NSCLC)的小，Travis^[14]^报道约1/2的BPC≤2 cm，Yi等^[15]^研究认为AC的大小比TC要大；本组病例平均为38.75 mm，其中TC的中位大小30.00 mm，而AC的为42.50 mm，AC的肿块似乎更大，但统计学比较两者无差异，可能与病例数少有一定的关系。本研究中央型占64.3%(18/28)，与Rosado de Christenson^[16]^报道一致。Gustafsson等^[17]^报道了流行病学登记调查的5 123例支气管类癌患者，59.0%肿块发生于右肺，本研究中66.7%(18/27)的肿块位于右肺。Marty-Ane^[18]^及Yi等^[15]^研究表明AC比TC表现为周围型肿块更常见，本研究中TC中外周型占31.25%(5/16)，AC中外周型占41.67%(5/12)，由于BPC起源于支气管及细支气管粘膜上皮及粘膜下腺体的kulchitsky细胞，越靠近中央，K细胞分布增多，所以大多数BPC都是中央型，中央型一般更加容易表现出临床症状，从而更加容易发现。

### 3.2 免疫组化指标

Ki-67与Bcl-2为常用的预测肿瘤复发与预后的指标。增殖细胞存在Ki-67的表达，目前已经很多研究表明Ki-67高表达提示预后较差^[10-12]^。Costes等^[10]^研究表明在TC中少于1%的核阳性，而AC中明显高于TC，核阳性为2.43%；一项包含31例类癌研究中(TC 21例，AC10例)^[19]^：28例BPC患者Ki-67的表达少于1%，而AC明显的Ki-67核阳性在(10%-20%)之间，Rugge等^[12]^研究表明，Ki-67的核阳性率在2.56%左右，AC的表达明显高于TC，并且认为以核阳性率5.4%为分界鉴别是否复发的敏感性与特异性分别为83%和97%，是BPC预后的独立的因素。本研究中Ki-67总体核阳性表达的中位数为2%，相关性分析表明的Ki-67的表达AC明显高于TC(*P*=0.033)，与核阳性表达是否大于5%为界，行单因素分析，结果示Ki-67高表达在总体及20例行肺癌根治术后的患者中为不良预后因素。

Bcl-2是提示细胞程序性死亡失调的指标，在BPC中，Bcl-2抗凋亡蛋白与Bax调亡蛋白前体相互表达，一般Bcl-2低表达占优势，从而可以促进细胞凋亡^[20]^，如果Bcl-2高表达占优势无法促进细胞凋亡，有研究^[11]^支持Bcl-2的过度表达与淋巴结的转移及复发相关。Rugge等^[12]^研究认为其平均阳性率在2.54%，在TC中的表达明显低于AC。本研究利用Bcl-2的表达用阳性与阴性表示，总体阳性率为22.3%，TC及AC分别为8.3%、50%，TC的阳性率明显低于AC(*P*=0.043)相关性分析Bcl-2与肿瘤复发有统计学差异(*P*=0.045)，但单因素分析显示Bcl-2并不是预后因素，可能和病例数较少有关。

TTF-1在类癌中的表达尚存争议。Rugge^[12]^认为TTF-1在支气管类癌中不表达。Sturm等^[21]^认为以前的研究TTF-1主要在神经内分泌癌，大多数是大细胞类癌，而被误解为在AC中表达。然而Folpe等^[22]^研究TTF-1在TC的阳性率为35%，AC中阳性率为75%，与Oliveira^[23]^报道一致。本研究中TTF-1在TC和AC中的阳性率均为40%，两者无统计学差异，因此我们认为其可以在BPC中表达，具体可否作为BPC的免疫组织化学指标有待进一步的研究。

许多研究^[5, 6]^表明大多BPC可以被CK染色成阳性，本研究中CK总体阳性率为88.5%，在TC与AC中的阳性率分别为86.7%，90.9%，两者无统计学差异，主要用于排除上皮性来源的肿瘤。有研究表明在TC中CgA、Syn、CD56阳性率比在AC中更高，本研究未发现此差异。

### 3.3 治疗与预后

BPC是一种预后较好的肿瘤，淋巴结转移率相对较低，Fink等^[1]^报道BPC的5年及10年生存率分别为88%和81%，TC的区域淋巴结转移率为3%-20%，AC为48%-75%，其他报道^[3-5]^TC的5年及10年生存率分别为90%-98%和82%-95%，而AC的预后明显差于TC，分别为61%-63%和35%-59%。最近一项西班牙的多中心研究García-Yuste^[24]^报道：TC和AC的5年生存率分别为97%、78%。在TC中淋巴结转移的发生率为9.1%(52/569：32例N1和20例N2)，在TC中为35.8%(33/92：14N1和19N2)，TC与AC中N2/N1的比例分别为1.36及0.63。本研究中TC的淋巴转移率为25%，AC为50%。病理为Ⅰ期的患者占53.4%(15/28)，其中TC的为62.5%(10/16)，AC为41.7%(5/12)。最近公布的一项大型研究^[14]^IASLC数据Ⅰ期的患者占82%(323/392)，SEER数据为78%(1 128/1 437，两组数据单因素分析比较均认为Ⅰ期与Ⅱ期的预后有统计学差异。本研究也得到了类似结果，Ⅰ期与Ⅱ期的患者预后有统计学差异(*P*=0.01)，本研究Ⅳ期占21.4%(6/28)，而IASLC数据Ⅳ期只有1例患者(1/392)，SEER数据为3%(49/1 437)。但相关数据没有区分BPC的亚型，所以无法对其亚型的特征进行对比性分析。而在另一个区分TC和AC的大规模研究^[25]^中，TC Ⅰ期的患者占87.35%(497/569)，AC Ⅰ期的患者占57.6%(53/92)。表明BPC发现时，不同于肺癌的主要类型(鳞癌和腺癌)，Ⅰ期的患者占大多数，并且在TC中所占的比例比AC大，可能与其有较好的预后也有一定的关系，BPC存在淋巴结的转移，病理分期是BPC患者的预后因素。

手术治疗仍然是支气管肺类癌主要的治疗方法，常规的肺叶切除及纵隔淋巴结清扫为标准术式，部分病例可以选择袖状切除以避免全肺切除。20例肺癌根治性手术的患者，总体5年生存率为73%，TC的5年生存率为83%，AC为57%。单因素分析，病理亚型(TC/AC)(*Log-rank*, *Mantel-Cox*, *P*=0.013)、术后有无淋巴结转移(*P*=0.004)进行肺癌根治性手术患者的预后因素；所以TC推荐行纵隔淋巴结清扫，AC要求纵隔淋巴结清扫。García-Yuste等^[24]^回顾性分析了304例(TC 261例，AC 43例)类癌未常规行纵隔淋巴结清扫的患者，并在357例(TC 308例，AC 49例)肺类癌行根治性纵隔淋巴清扫的前瞻性研究，认为根治性淋巴结清扫明显改善AC的预后。本研究中，TC的预后明显好于AC，BPC亚型和有无淋巴结转移，是影响预后的因素。
